# Clinical therapeutic effects of platelet-rich plasma in patients with burn wound healing: A protocol for systematic review and meta-analysis

**DOI:** 10.1097/MD.0000000000026404

**Published:** 2021-08-06

**Authors:** Jie Li, Wang-Ping Hu, Guo Zhong

**Affiliations:** a Department of Emergency Surgery, Tianyou Hospital Affiliated to Wuhan University of Science & Technology, Wuhan, Hubei, China; b Department of Critical Care Medicine, the First People's Hospital of Jiangxia District, Wuhan, Hubei, China.

**Keywords:** burns, meta-analysis, platelet-rich plasma, wound healing

## Abstract

**Background:**

In clinical settings, burn wounds are frequently encountered. Since burn wounds are a form of physical injury, they can have long-term adverse effects on the human body. It has been a significant challenge to treat burn wounds completely. Since traditional treatment strategies have been unable to heal burn wounds completely, they lack the efficacy to cure the wounds without long-term effects, such as heavy scarring. Reportedly, platelet-rich plasma (PRP) has shown potential to accelerate wound healing. Yet, there are no conclusive reports on a methodological comparative study of research that has assessed the medical benefits of PRP for treating individuals carrying burn wounds. Thus, the present meta-analysis and systematic study aims to assess the medical benefits of PRP for treating patients carrying burn wounds.

**Methods:**

The authors will conduct a comprehensive search for randomized controlled trials that evaluate the safeness and efficiency of PRP to treat burn wounds. The search includes 3 Chinese language databases (WanFang database, Chinese BioMedical Literature database, and China National Knowledge Infrastructure) and 4 English language databases (Cochrane Library, EMBASE, Web of Science, and MEDLINE). These electronic databases will be searched from their establishment till May 2021. A pair of independent authors will be selecting eligible studies for extracting data. The same authors will employ the Cochrane risk of bias tool to evaluate the bias risk. We will make use of RevMan (version: 5.3) software to complete data synthesis.

**Results:**

The present protocol will establish practical and targeted results evaluating the efficacy and safeness of using PRP to treat burn wounds. The current study also provides a reference for clinical use of PRP.

**Conclusion:**

Stronger evidence about the effectiveness and safety of using PRP to treat and heal burn wounds will be provided for clinicians to refer.

**Ethics and dissemination:**

Ethics approval is unrequired.

**Registration number:**

March 31, 2021.osf.io/whauj. (https://osf.io/whauj/).

## 1 Introduction

Burn wounds are among the list of highly debilitating body wounds. Burn wounds are widely related to both physical and emotional damage.^[1]^ Moreover, burn wounds also inflict adverse outcomes on several organs and cause significant levels of morbidity and fatalities.^[2,3]^ Reportedly, each year, nearly 30 million worldwide fatalities occur through burn wounds.^[4]^ Unlike other injuries, burn wounds inflict scarring and disfigurements in patients even after healing.^[5,6]^ Currently, there are numerous therapeutic strategies to treat burn wounds, including autografting, xenografting, and using bioengineered skin substitutes. However, numerous clinical limitations are associated with these methods.^[7]^ Consequently, finding effective treatment strategies to completely treat burn wounds is still a significant challenge in the health sector.

Wound healing is an active process with several phases. There is a synchronized effort from various biological pathways. In general, wound healing is separated into swelling, angiogenesis, proliferation, and final phase of advancing. An intricate network of signals between different types of cells is formed during the different stages of tissue healing, which includes cytokine secretion, development factors, and collagen matrices.^[8]^ Reportedly, platelet-rich plasma (PRP) has been used as a form of treatment for a range of diseases with positive outcomes, which includes surgery, knee osteoarthritis, diabetic ulcers, etc.^[9–11]^ Recent researches involving PRP have shed some light on its potential clinical application to treat burn wounds. Yet, there haven’t been any attempts to logically assess the medical benefits of PRP for treating and healing burn wounds. Therefore, the present protocol will examine the medical effects of using PRP to treat and heal patients carrying burn wounds.

## 2 Methods

### 2.1 Study design and registration

This study has been registered in the OSF (http://osf.io/): DOI 10.17605/OSF.IO/WHAUJ. The design of this study adheres to The Preferred Reporting Items for Systematic Review and Meta-Analyses Protocols statement guidelines.

### 2.2 Inclusion criteria for study selection

#### 2.2.1 Types of studies

The authors will include all randomized controlled trials describing the clinical therapeutic effects of PRP for burn wound healing.

#### 2.2.2 Types of participants

Participants include those clinically diagnosed with burn injuries, all participants will be included, regardless of the depth and site of the burn wounds.

#### 2.2.3 Types of interventions

We will consider studies that compare PRP with placebo, no intervention, saline, or any other wound dressings. Articles that utilize PRP in combination are unable to objectively assess the clinical therapeutic effects of PRP, therefore, such articles will be rejected.

#### 2.2.4 Types of outcome measures

The primary outcome will include the healing rate of burn wounds. The secondary outcomes will include allergic reactions, inflammatory markers, collagen deposition graft loss, and related adversities.

### 2.3 Search methods for the identification of studies

#### 2.3.1 Electronic searches

The authors will conduct a comprehensive search for randomized controlled trials that evaluate the safeness and efficiency of PRP to treat burn wounds. The search includes 3 Chinese language databases (WanFang database, Chinese BioMedical Literature database, and China National Knowledge Infrastructure) and 4 English language databases (Cochrane Library, EMBASE, Web of Science, and MEDLINE). These electronic databases will be searched from their establishment till May 2021. The present study does not pose restrictions on status of publication or language. The following key terms will be used for the literature search, which includes the following domains: “platelet-rich plasma,” “platelet rich,” “platelet plasma,” “platelet gel” paired with “burn∗.” The terms will be combined with “OR” or “AND.”

#### 2.3.2 Searching other resources

The authors will conduct another search on ClinicalTrials.gov (https://clinicaltrials.gov/) and Google Scholar to look for additional related studies and ongoing clinical trials related to PRP and burn wound healing in grey literature.

### 2.4 Data selection and analysis

#### 2.4.1 Selection of studies

**Figure 1:**
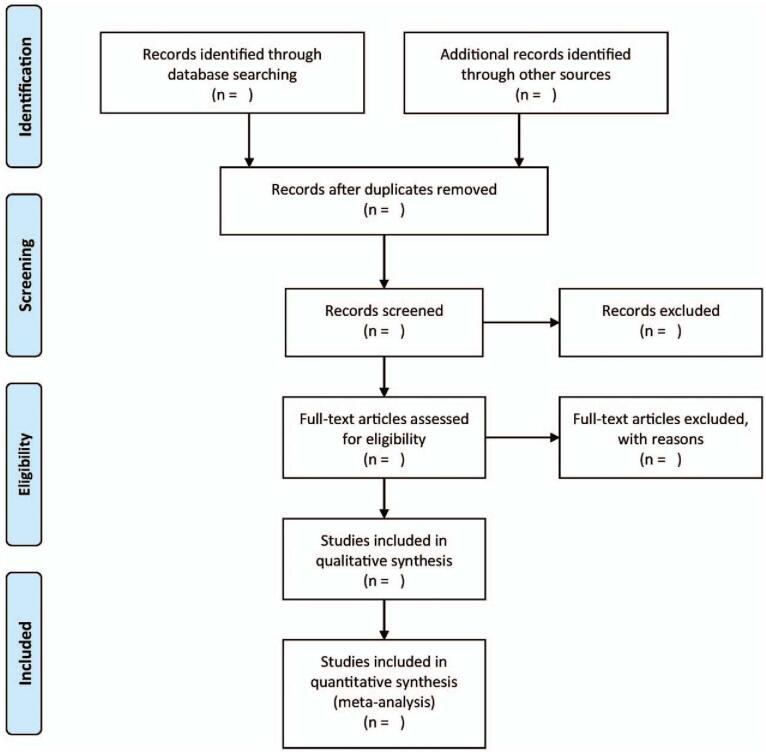
Flowchart of study selection process.

A pair of independent authors will be screening the search results by going through the titles/abstracts. All instances of duplicate studies and reviews will be rejected. Upon identifying potential studies, the authors will study the complete text of the shortlisted studies to evaluate if the studies satisfy the criteria for inclusion. All differences in opinions between the 2 authors are sorted by consulting with a separate author. Figure 1 presents the process related to selecting studies.

#### 2.4.2 Data extraction and management

A general form for collecting study data will be created before extracting data, which will be based on the inclusion criteria. The extracted data will include the following information: the first author, publication date, ethnicity, country, study design, inclusion and exclusion criteria, sample size, detailed interventions, follow-up period. All differences in opinions between the 2 authors are sorted by consulting with a separate author.

#### 2.4.3 Assessment of risk of bias

A couple of independent authors will adopt the Cochrane risk of bias assessment tool to estimate the bias risk.

#### 2.4.4 Measures of treatment effect

In the case of continuous outcomes, the authors will represent the outcomes as mean differences or standardized mean differences with 95% confidence intervals to calculate the treatment effect. In the case of dichotomous data or categorical data, the authors will make use of the relative risk with 95% confidence intervals to determine the treatment effect.

#### 2.4.5 Assessment of heterogeneity

We will use Cochran *Q* statistic and *I*^2^ statistic to determine the heterogeneity between the study results. In the event where the heterogeneity is large, we will adopt the random-effects model for the analysis. Else, we will use the fixed-effects model.^[12,13]^

#### 2.4.6 Assessment of reporting biases

The authors will use Egger approach to assess the funnel plot asymmetry for reporting biases and small-study effects.

#### 2.4.7 Sensitivity analysis

The authors intend to conduct a sensitivity analysis to evaluate the reliability and robust condition of the findings in the present study.

## 3 Discussion

In the past, clinical trials have indicated that PRP has a significant role in treating and healing burn wounds. However, there has been no systematic review that has conclusively evaluated the medical benefits of PRP in the context of healing burn wounds, and studies are still at the theoretical standard. Taking into consideration the abundant literature on PRP for treating burn wounds, the authors plan to conduct a systematic review to establish the medical benefits of PRP when used to treat and heal burn wounds. It is expected that the findings of the present systematic analysis would provide up-to-date evidence regarding the medical benefits of using PRP to treat and completely heal patients with burn wounds. Moreover, the conclusions of this meta-analysis could be a useful reference clinicians, patients, and health policymakers.

## Author contributions

**Conceptualization:** Jie Li, Wang-Ping Hu.

**Data curation:** Jie Li, Wang-Ping Hu.

**Formal analysis:** Jie Li, Wang-Ping Hu.

**Funding acquisition:** Jie Li, Guo Zhong.

**Investigation:** Jie Li, Wang-Ping Hu, Guo Zhong.

**Methodology:** Wang-Ping Hu, Guo Zhong.

**Resources:** Jie Li, Guo Zhong.

**Software:** Jie Li.

**Validation:** Jie Li, Wang-Ping Hu, Guo Zhong.

**Visualization:** Jie Li, Wang-Ping Hu.

**Writing – original draft:** Jie Li, Wang-Ping Hu.

**Writing – review & editing:** Guo Zhong.
